# Health-Promoting Potential of the Mediterranean Diet and Challenges for Its Application in Aging Populations

**DOI:** 10.3390/nu17233675

**Published:** 2025-11-24

**Authors:** Marta Cianciabella, Stefano Predieri, Rachele Tamburino, Chiara Medoro, Roberto Volpe, Stefania Maggi

**Affiliations:** 1 Institute for BioEconomy, National Research Council (CNR), Via Piero Gobetti 101, 40129 Bologna, Italy; marta.cianciabella@cnr.it (M.C.); stefano.predieri@cnr.it (S.P.); rachele.tamburino@cnr.it (R.T.); chiara.medoro@cnr.it (C.M.); 2 Health and Safety Unit (SPP), National Research Council (CNR), Piazzale Aldo Moro, 7, 00185 Roma, Italy; roberto.volpe@cnr.it; 3 Neurosci Institute (IN), National Research Council (CNR), 35128 Padua, Italy; stefania.maggi@in.cnr.it

**Keywords:** Mediterranean Diet, health, elderly, bioactive molecules, sensory

## Abstract

The Mediterranean Diet (MD) is a lifestyle that involves not only dietary habits, well known for their effectiveness in preventing health risks by supplying well-balanced foods rich in bioactive compounds, but also daily habits that improve the quality of life. Older adults represent a segment of the population that can particularly benefit from this dietary pattern. However, the specific characteristics and needs of older individuals require a critical analysis of aspects that may limit adherence to the MD principles, including physical impairments related to eating, sensory and cultural aspects, accessibility of food sources, and the social context. The objective of this study was to review the potential benefits of the MD in relation to the needs, capacities and eating behaviors of older adults, focusing on the beneficial effects of plant-based food metabolites and their suitability for older adult diets. The results demonstrate how the MD can be tailored to meet the nutritional and functional needs of older adults, supporting healthy aging. Therefore, the Mediterranean lifestyle could be an effective tool in public health policies to promote healthy habits, thereby improving the quality of life in vulnerable population categories.

## 1. Introduction

The MD is a dietary lifestyle that emerged from studies conducted in the early 1950s by Ancel Keys and his team on the eating habits of countries bordering the Mediterranean Sea [1]. The original MD has undergone significant changes over time, influenced by shifts in culture, society, economy, and work-related transformations, leading to a reduction in adherence to its core principles [2]. Nevertheless, the benefits of such a dietary regimen in maintaining health, preventing communicable diseases, and improving longevity are widely recognized [3,4,5]. In 2013, MD was inscribed on the UNESCO Representative List of Intangible Cultural Heritage of Humanity (UNESCO). The MD is characterized by a high consumption of fresh or dried fruits and vegetables, legumes, and whole-grain cereals. It also includes moderate consumption of fish and dairy products and a limited intake of red meat. Olive oil represents the main source of fat [6]. These features make the MD particularly suitable for promoting healthy aging [7]. Moreover, it can help mitigate age-related diseases in later life [8] and counteract the onset of frailty [9]. A greater adherence to a plant-based diet, studied a posteriori in a group of European elders, is associated with lower all-cause mortality [10], indicating the relevant impact of lifelong habits. However, actions aimed at maintaining or possibly increasing adherence to MD at a later age can positively affect health status and survival expectations. The correlation between health and adherence to balanced diets is well established across all stages of life but becomes particularly significant in later life due to its impact on healthy aging [11]. Finally, the MD adoption stimulates respect for natural resources and seasonality, playing an important role in preserving the environment. Indeed, the MD has been demonstrated to be the most sustainable diet considering several factors such as the environment, nutrition, the economy and socio-cultural aspects [12].

In 2025, the Italian National Institute of Health published the Italian MD Guidelines [13], developed based on the systematic reviews and meta-analyses of 3839 clinical studies, involving approximately 2 million participants, followed for an average of 13 years. The guidelines are based on 108 evidence-based recommendations on how the MD can help in disease prevention and management. Key outcomes supported by research include the possibility of lowering overall and cardiovascular mortality, reducing cancer incidence, and improving metabolic and cognitive health, with remarkable positive effects on the aging process. The guidelines underline the fact that the MD is recognized not only as a dietary pattern but as a comprehensive lifestyle that promotes health, prevents chronic diseases, and supports environmental sustainability in terms of natural resource consumption and environmental impact. It emphasizes not only balanced, mostly plant-based nutrition, but also regular physical activity and rest, conviviality, consumption of seasonal and local foods, short supply chains and minimally processed ingredients. Finally, a key aspect of the guidelines also concerns the economic impact of the MD. Several studies suggest that adopting this dietary pattern could significantly reduce healthcare costs, particularly at older age.

This review aims to highlight the scientific foundations underlying the beneficial effects of the MD, while also exploring its cultural and social implications. Although several reviews are available on the MD’s advantages, the present work addresses a specific gap in the literature by providing a comprehensive overview of the main benefits associated with its adoption, focusing on both the metabolites derived from dietary intake and the socio-cultural aspects that underpin the healthy lifestyle, which could enhance well-being in vulnerable population groups such as older adults in later life.

## 2. Methods

This narrative review investigates the health benefits of the MD on a specific population group, the older adults, based on the evidence currently available in the literature. The narrative approach was chosen due to the complexity and multidimensional nature of the topic. The literature search was carried out using multiple databases, including Web of Science, PubMed, Scopus and Google Scholar between November 2024 and October 2025. The primary search terms included “Mediterranean Diet” and “aging,” along with combinations of words such as “Mediterranean Diet + sustainable,” “Mediterranean Diet + healthy aging + bioactive compounds,” and “dietary + lifestyle + aging.” The previous terms were also used in combination with specific food categories. The search was limited to English-language publications, giving priority to papers from the past ten years to ensure the inclusion of the most recent and relevant studies. However, older references were also considered when updated data were not available.

Additional articles were identified from the references cited in the original papers. The selection process involved several stages: initial screening based on titles and abstracts, followed by full-text analysis. The following exclusion criteria were applied to the search: (a) inappropriate topics, not pertinent to the specific themes addressed in each section of the review, and (b) PhD dissertations, conference proceedings, abstracts, and unpublished studies. Finally, a total of 178 articles were selected for the review.

In the section regarding the MD bioactive molecules, both human studies (prospective cohorts, randomized trials, and meta-analyses) and translational or mechanistic research were included when they contributed to understanding the biological plausibility of observed health effects.

## 3. MD for Aged People

The aging process involves multiple events during which cells are stressed by endogenous and exogenous elements, causing DNA damage, mutations, dysfunctional protein accumulation (heat shock protein), oxidative stress, mitochondrial dysfunction and inflammation [14]. The phytochemical compounds, minerals and vitamins present in MD food can counteract these processes, promoting healthy aging and longevity, and slowing down the aging process by acting as antioxidants and/or anti-inflammatories [15,16].

### 3.1. MD in Disease Prevention

Adherence to MD can contribute to preventing and/or mitigating the main diseases affecting older people, such as diabetes, cardiovascular diseases, and cancer [4,17]. Indeed, evidence on the effectiveness of the MD against the most common chronic diseases is constantly growing [12].

MD provides defense against cardiovascular diseases in both the general population and in patients already affected by these diseases. Moreover, it has been reported that the MD adherence decreases the risk of heart attacks, various types of coronary artery disease, stroke, and cardiovascular mortality [18].

There is a direct association between the MD pattern and the reduced risk of diabetes. Particularly, it has been shown that even modest adherence to the MD likely decreases the incidence of type 2 diabetes [19].

Recently, the European Prospective Investigation into Cancer and Nutrition conducted a study in 23 centers of 10 European countries, declaring that the MD exerts protective action against the four most frequent cancers in the European population (colorectal, breast, lung, and prostate cancer). High consumption of fruit, vegetables, fish and yogurt, coupled with low consumption of alcohol and red and processed meat, lowers the risk of cancer development [20].

### 3.2. MD in Comorbidities Prevention

Mental decline and dementia are the main comorbidities and among the most common causes of death in the elderly [12]. Following the MD lifestyle lowers the risk of cognitive impairments, improving mental health [20] and protecting against mental illnesses such as Alzheimer’s disease [21,22]. Indeed, phytochemical compounds present in Mediterranean food are active against oxidative stresses, protecting the brain and the nervous system from inflammation and the accumulation of AB plaques [23]. Other studies found adherence to MD effective in mitigating depression, anxiety and psychological distress [24].

Following MD can also support physical performance maintenance, counteracting the age-related muscle mass and mineral bone density reductions, preserving sexual capacity, and preventing immune system dysregulation [11].

### 3.3. MD in Sarcopenia Prevention

Sarcopenia is a neuromuscular degeneration caused by loss of physical function, particularly in older adults. Its progression can lead to various dysfunctions such as metabolic, osteoarticular, and cognitive disorders, which may result in malnutrition, physical inactivity, falls, depressive symptoms, and death [25]. Nutrition and dietary interventions are effective approaches to treat and prevent sarcopenia. The MD lifestyle positively influences physical function and helps prevent sarcopenia through typical foods such as extra-virgin olive oil, fruits, vegetables, and fish, and their anti-inflammatory and antioxidant properties, as well as the promotion of a healthy lifestyle that includes social and physical activity [26]. In fact, recent recommendations to prevent sarcopenia involve reducing sedentary time and increasing protein intake above the RDA (0.8 g/kg/day) to mitigate muscle loss [27], since sarcopenic older adults tend to consume less protein [25]. Accordingly, a total daily protein intake of around 1.6–1.8 g/kg/day, with three main meals containing 0.6 g/kg of high-quality protein sources such as wheat protein—particularly abundant in the MD [28]—is an effective strategy to optimize nutrition and maintain muscle mass during aging [29]. MD meets these recommendations, aligning sensory satisfaction, palatability, and protein distribution across meals, promoting healthy nutrition.

### 3.4. MD Adherence in Later Age

Only if the adherence to the MD has a consistent and foundational presence in daily eating habits and is supported by both food education and eating pleasure do the recognized health benefits have the potential to become effective. Interventions aimed at enhancing this aspect should align with the statement, ‘Make “good for you” taste good’ [25]. This means exploring and respecting individual choices and preferences. In this context, formulating suitable dietary strategies for older adults should consider their longstanding adherence to traditional eating practices but also their preferences for heritage eating habits [26]. According to this research, a convenient strategy is to explore alternative ingredients that enhance the health properties of traditional foods while maintaining most of their overall characteristics. Incorporating elements of MD into commonly consumed foods represents a promising approach that enhances their nutritional profile without compromising consumer acceptance. Indeed, Albergamo et al., in 2021 [27], reformulated chicken burgers by incorporating typical MD ingredients, such as tomato, rosemary, basil, and thyme, together with powdered fortifying agents. A consumer test conducted with older adults demonstrated significantly higher levels of appreciation for the reformulated product compared to the standard burger. A similar approach was adopted by offering older adults’ fish-based sausages enriched with various combinations of vegetables and herbs [28]. In this case, senior consumers actively contributed to the development of acceptable recipes. A semi-trained panel of older adults evaluated each step of the product innovation process. This led to the creation of a healthy and nutritious novel food, well appreciated by older consumers. MD can also drive the formulation of the “Mediterranean” version of traditional foods, satisfying old people’s food habits while improving the healthiness of a typical recipe. Volpe et al., in 2021 [29], involved aged consumers in the evaluation of MD-based pasta sauces and lasagna fillings as alternatives to red meat-based Bolognese ragù, showing that tailored-taste alternative ingredients can match the appreciation level of standard meat-based dishes.

## 4. Mediterranean Food Plants and Bioactive Molecules

**Figure 1:**
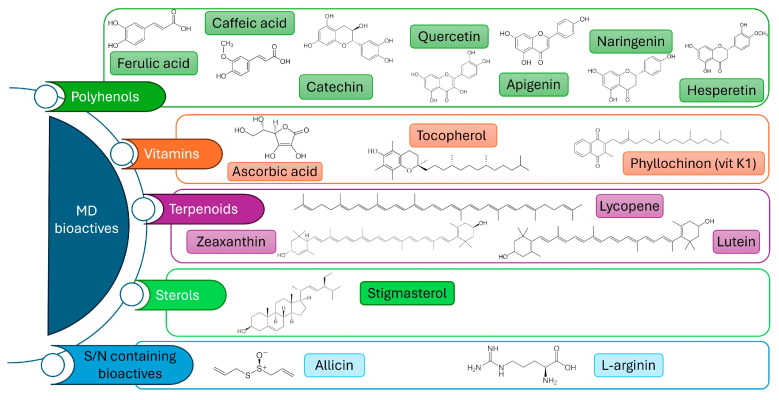
Chemical structures of selected bioactive compounds found in plants characteristic of the Mediterranean diet. These include, but are not limited to, polyphenols, vitamins, terpenoids, sterols, S- and N-containing compounds, and phenolic acids, all of which contribute to the health-promoting properties of the diet. The structures are shown to illustrate the diversity of molecular classes derived from edible plants native to the Mediterranean region.

The Mediterranean Diet model is grounded in a diverse range of foods with the predominance of plant-based foods, including fruits, vegetables, legumes, nuts, whole grains, and extra-virgin olive oil, which contribute to macronutrient balance, essential micronutrients and a diverse array of bioactive compounds, key mediators of the beneficial health outcomes associated with the MD. Bioactive molecules are non-nutrient secondary metabolites that exert regulatory effects on human physiology. In the context of the MD, the most relevant categories include polyphenols, carotenoids and terpenoids; vitamins with antioxidant properties; bioactive peptides; and sulfur-containing compounds (Figure 1). These compounds have been shown to modulate key biological processes linked to age-related pathologies, including oxidative stress, dysregulated lipid metabolism, endothelial dysfunction, and impaired immune responses. By targeting these mechanisms, they contribute to the prevention of cardiovascular and metabolic diseases, the maintenance of cognitive function, and the promotion of healthy aging in the elderly.

Whole grains such as wheat, rye, barley, and oats deliver alkylresorcinols (i.e., phenolic lipids that serve as biomarkers of whole-grain intake), β-glucans (i.e., soluble fibers), phenolic acids (e.g., ferulic, caffeic, and p-coumaric acids), flavonoids, lignans, tocols (i.e., vitamin E isomers), and phytosterols. Evidence from large prospective cohorts and meta-analyses supports their role in reducing the incidence of cardiovascular disease and type 2 diabetes in older adults [30,31,32]. These compounds modulate lipid and glucose metabolism, attenuate systemic oxidative stress, and exert vasculoprotective actions [33,34,35,36,37]. Mechanistic and translational studies further indicate favorable interactions with gut microbiota, enhancing the production of short-chain fatty acids and thereby influencing immune function, processes central to healthy aging [38]. Processing and cooking strongly influence the bioavailability. Milling into refined flour removes bran and germ, drastically reducing fiber, phenolic acids, and phytosterols [39], whereas thermal treatments can degrade heat-sensitive tocols and flavonoids but may also increase the release of bound phenolic acids, enhancing colonic bioaccessibility after digestion [38,40]. Overall, according to the most relevant geriatric outcomes, the strength of the evidence is high, as the reported associations are consistently supported by findings from multiple cohort studies and randomized intervention trials (Table 1).

Legumes provide not only plant protein (15–40% g/100 g dry basis depending on the species [64,65,66]) and fermentable fiber (~12% g/100 g dry basis [67,68]) but also secondary metabolites such as flavonoids (e.g., catechin, epicatechin, quercetin, quercetin-3-O-glucoside, myricetin, kaempferol-3-O-rutinoside, and kaempferol-3-O-glucoside), especially abundant in legumes with colored seed coats, condensed tannins, saponins and tocopherols (δ- and γ-isoforms) [69,70]. These molecules have demonstrated antioxidant, antihypertensive, hypocholesterolemic, and anticancer activities, contributing to the prevention of sarcopenia and immune decline, conditions often exacerbated in later life [66,71,72,73,74,75,76,77]. Recent systematic reviews and meta-analyses indicate that higher legume intake is associated with reduced all-cause mortality and improved metabolic outcomes [42,43]. In addition, bioactive peptides and small proteins (e.g., lunasin, Bowman-Birk inhibitors) exert antioxidant, immunomodulatory and anti-inflammatory activities [78,79,80]. Overall, according to the most relevant geriatric outcomes, the evidence can be considered moderate, as it is supported by consistent observational data and several intervention studies primarily addressing metabolic outcomes rather than long-term geriatric endpoints (Table 1).

Tomato, one of the most popular and consumed components of MD, is a unique source of bioactive molecules such as lycopene, polyphenols, phytosterols, and polyamines. Lycopene accumulates in the prostate, brain and vascular tissues, where it reduces oxidative DNA damage and supports cellular homeostasis [46]. Observational meta-analyses suggest an inverse association between tomato/lycopene intake and cardiovascular outcomes [45]. However, a recent meta-analysis of interventional trials [44] found no consistent improvements in established risk factors such as blood pressure or lipids, highlighting the need for caution in extrapolating mechanistic findings to clinical outcomes. Processing strongly influences lycopene bioavailability. Heat treatment reduces vitamin C and some phenolics, but it also disrupts the plant cell matrix and enhances lycopene bioavailability, especially in the presence of olive oil [81,82]. In addition, tomatoes provide polyamines (e.g., spermidine, putrescine, and spermine), which have been recently linked to longevity [83], and phytosterols (e.g., β-sitosterol, campesterol, stigmasterol) with cholesterol-lowering effects and improvement of cardiovascular outcomes, which is highly relevant in older adults [84], and flavonoids such as quercetin, naringenin, and chlorogenic acid [85]. Tomato by-products, such as peels and seeds, also provide concentrated sources of carotenoids, polyphenols, dietary fiber, and anthocyanins, particularly in pigmented varieties, making them valuable raw materials for functional food formulations [86,87]. Finally, water-soluble compounds in tomatoes, including nucleosides, nucleotides, and phenolic acids, exhibit antiplatelet activity, thus showing antithrombotic effects [88,89]. Overall, according to the most relevant geriatric outcomes, the evidence is moderate, supported by findings from cohort studies and mechanistic research, although most interventional trials remain short-term (Table 1).

Leafy vegetables such as spinach (*Spinacia oleracea*), kale (*Brassica oleracea*), lettuce (*Lactuca sativa*), chard (*Beta vulgaris*), arugula (*Eruca sativa*), and chicory (*Cichorium intybus*) and cucurbits such as zucchini (*Cucurbita pepo*), pumpkin (*Cucurbita moschata*), cucumber (*Cucumis sativus*), and melon (*Cucumis melo*) provide high levels of carotenoids like lutein and zeaxanthin, folates, vitamin K1, glucosinolates, and saponins and dietary fibers [47,90,91,92,93]. Carotenoids are critical for visual health, particularly in the prevention of age-related macular degeneration, while folate status has been consistently linked to cognitive performance in older adults [48]. Vitamin K1 contributes to vascular, skeletal and neural protection [90]. Members of the Brassicaceae family, particularly kale, supply glucosinolates, precursors of isothiocyanates, which have been shown to regulate detoxification enzymes and exert anticancer effects [94]. Saponins can reduce intestinal cholesterol absorption, modulate immune responses, and display antitumor effects [75,95,96]. Finally, both leafy greens and cucurbits are excellent sources of dietary fiber, which supports gut microbiota diversity, improves glycemic control, lowers cholesterol, and may reduce colorectal cancer risk [31]. Overall, according to the most relevant geriatric outcomes, the evidence is moderate, with high epidemiological consistency, although data from long-term intervention studies in older populations remain limited (Table 1).

Citrus fruits supply vitamin C, flavanones (e.g., hesperidin/hesperetin and naringin/naringenin), polymethoxylated flavones (nobiletin, tangeretin), and carotenoids [49,50,51]. Meta-analyses report improved endothelial function and reduced risk of cardiovascular events [49]. Importantly, recent evidence highlights the role of citrus flavanones, including neohesperidin, hesperidin, and hesperetin, in vascular, cognitive and skeletal health. While mechanistic studies and short-term interventions support antioxidant, anti-inflammatory, and anti-apoptotic activities, clinical outcomes remain heterogeneous. In a 36-week randomized, placebo-controlled trial in older adults with subjective cognitive decline, citrus peel extract supplementation showed no significant improvement over placebo, underscoring the need for cautious interpretation of translational evidence [53]. Overall, according to the most relevant geriatric outcomes, the evidence can be considered moderate, showing consistency across human trials and mechanistic studies, although some results remain neutral. (Table 1).

Allium species, especially garlic, are widely recognized for their rich content of bioactive phytochemicals. Among these are sulfur-containing compounds (thiosulfinates, allicin, diallyl sulfides, S-allyl cysteine) with antimicrobial, antihypertensive, and cardioprotective activities [97,98,99]. Meta-analyses consistently demonstrate that garlic supplementation can lower blood pressure and modestly reduce cholesterol in hypertensive adults [54,55]. Recent randomized trials also show the benefits of aged garlic extract on arterial stiffness and gut microbiota in hypertensive individuals [56]. Phenolic compounds, including β-resorcylic acid, pyrogallol, protocatechuic acid, gallic acid, rutin, and quercetin, further enhance antioxidant protection [98]. Overall, according to the most relevant geriatric outcomes, the evidence is moderate, supported by meta-analyses of randomized controlled trials, although some heterogeneity remains across studies. (Table 1)

Nuts, including almonds, walnuts, pistachios, and hazelnuts, are increasingly recognized as functional foods thanks to their richness in bioactive molecules. They provide a balanced profile of unsaturated fatty acids, including both monounsaturated (MUFA) and polyunsaturated fatty acids (PUFA). Also, nuts supply tocopherols, especially vitamin E, found in high concentrations in almonds and hazelnuts; phytosterols; polyphenols (e.g., walnuts are particularly rich in ellagitannins, while almonds contain relevant amounts of flavan-3-ols); L-arginine; and minerals such as magnesium [100,101]. Numerous meta-analyses and large cohort studies confirm the inverse association between nut consumption and cardiovascular and all-cause mortality, supporting a dose–response effect up to about 30 g/day [57,58,59]. Evidence remains strong for cardiovascular outcomes but moderate for cancer prevention. These findings align with the Mediterranean dietary pattern’s overall cardioprotective role [57,102,103,104,105,106,107]. Overall, according to the most relevant geriatric outcomes, the evidence is strong, confirmed by multiple large cohort studies and intervention trials (Table 1).

Beyond staple foods, Mediterranean cuisine incorporates a wide array of seasonings and minor vegetables that further enrich its bioactive profile. Caper (*Capparis spinosa* L.), widely used as seasoning, and Mediterranean herbs (oregano, rosemary, sage, thyme, basil, parsley) provide terpenoids, essential oils, and polyphenols such as rosmarinic and apigenin acids [60,62,63,108,109,110,111,112,113,114,115,116,117,118,119,120]. These compounds improve cerebral blood flow, modulate cholinergic neurotransmission, and reduce neuroinflammation, supporting cognitive and vascular health in older adults [60]. Overall, according to the most relevant geriatric outcomes, the evidence is limited, as most data derive from mechanistic or preclinical studies, and randomized trials are lacking (Table 1).

### Mediterranean Dietary Synergy

The MD represents a complex phytochemical network whose synergistic bioactive molecules directly target hallmarks of aging (e.g., oxidative stress, chronic inflammation, metabolic dysfunction, and neurodegeneration [121,122]). Evidence from large meta-analyses and prospective cohorts (MD overall, Table 2) consistently associates higher adherence to the MD with reduced all-cause mortality, cardiovascular disease, frailty and cognitive decline [123,124]. In addition, the NU-AGE one-year multicenter randomized controlled trial, conducted across five European countries, tested a personalized Mediterranean-like diet ensuring micronutrient adequacy and demonstrated improvements in gut microbiota composition, reductions in frailty, and favorable changes in immune and metabolic biomarkers related to inflammaging [125,126]. These findings provide mechanistic support for the biological plausibility of the MD’s benefits on healthy aging, although the generalizability remains limited by the high adherence and relatively healthy status of participants. Finally, polyphenol-rich dietary patterns, characterized by high consumption of fruits, vegetables, tea, cocoa, and red wine, have been systematically associated with reduced oxidative stress, inflammation, and vascular aging [105]. Overall, according to the most relevant geriatric outcomes, evidence for composite Mediterranean-style dietary patterns is strong, supported by multiple complementary study designs that together provide mechanistic, interventional, and epidemiological coherence (Table 2).

## 5. Sensory Perceptions and Food Preferences Among the Elderly

### 5.1. Sensory Perceptions in Aged People and MD

The aging process is accompanied by a decline in sensory acuity, as well as the onset of impairments affecting mastication, deglutition, and digestion [127]. When the perception of the sensory stimuli through the five senses is impaired by a physiological event, such as aging, the resulting loss and distortion of the sensory perception, paralleled by phenomena of sensory-specific satiety [128], can lead to less food enjoyment and, in turn, less appetite and food intake, resulting in malnutrition [129]. The elderly can face undernutrition due to some food category deficiencies, such as protein deficiency; thus, although the age-related reduction in sensory-specific satiety favors a more monotonous diet, it is essential to maintain the variety and the richness in the products they consume [130]. This highlights the need to design elderly-friendly MD foods addressing such challenges while preserving food appreciation [131]. Olfactory loss is one of the most common phenomena observed. However, it was observed that older adults affected by this impairment tend to increase their consumption of MD basic food items such as fruits and vegetables while reducing high-fat foods [132]. A growing tendency to adopt MD principles has also been documented among elderly individuals in Italy [133], with age-related increases in the intake of fruits, vegetables, and legumes, particularly pronounced among women.

### 5.2. The Sensory Science to Support Adherence to MD

Sensory science offers valuable methods for investigating older consumers’ attitudes and preferences and can support the development of satisfactory and palatable foods tailored to this population [134]. Further improvements can be achieved through advanced technologies that adapt food texture to older adults’ needs. Liu et al., in 2022 [131], reported that soft, smooth, and moist foods are more suitable for the elderly. However, despite the expected decrease in olfactory acuity, odorous stimuli should also be considered. A survey conducted on over 65 Italian consumers asked participants to indicate their preferred sensory features for a functional food. The most appealing attributes reported were pleasant odor and flavor, as well as soft, warm, liquid, and thick textures [135]. This co-creation approach, based on in-depth interviews integrated with on-site testing adapted to older adults’ skills [134], can support the introduction of MD foods appreciated by elderly consumers. The age-driven loss of sensitivity generally hurts eating pleasure, an essential component affecting food intake [136]. Nevertheless, a decreased sensitivity to bitterness with age may improve the appreciation of healthy vegetables such as Brassicaceae (e.g., cabbage, Brussels sprouts) and salad greens [137]. MD also includes a common use of herbs and spices, which can stimulate sensory perception and increase food appreciation while providing phenolic compounds and other bioactive substances [138,139]. Adherence to MD was reported to be correlated with aromatic plant consumption, but not with spices [140]. As reported by Laureati et al. in 2008 [141], the elderly show a high interest in foods with enhanced taste and flavor stimuli. Adding flavor enhancers to the cooked meals was found to be effective for improving dietary intake and body weight in elderly nursing home residents [142]. Mediterranean herbs, including parsley, oregano, dill, and rosemary, were effective when tested for improving flavor intensity and liking of protein-rich foods [143], although not clearly correlated to the protein intake. Spices may be effective in improving sensory perception, allowing the potential reduction of salt [144,145].

Visual stimuli can also help increase food appeal and appetite. Even observing simple food pictures can activate brain regions, near the gustatory areas, involved in taste perception [146]; moreover, the brilliant colors of fresh Mediterranean products may contribute to making food experiences more enjoyable for older people.

## 6. The MD Lifestyle as a Social Driver for Age-Friendly Communities

The MD was recently recognized as a healthy and sustainable dietary pattern [12] based on local seasonal food, conviviality, food sustainable production and lifestyle [147]. These principles can be particularly relevant for older adults, who may engage in post-retirement activities such as urban horticulture, providing multiple sensory stimulation [148,149] and home cooking [150], both of which offer recognized health benefits, especially for mental well-being. Horticultural activity in later life can contribute to preserving biodiversity and social sustainability, while also providing individuals with fresh, seasonal products. A garden can serve as a supplement to a self-subsistence strategy for fresh fruit and vegetables [151] and implement their consumption [152]. The MD encourages the cultivation of indigenous species, local cereals, fruit and vegetables, which help preserve the environment and its biodiversity, while sustaining the traditional knowledge of their use [153], usually passed down through gardening activities by the elderly. It has been reported that gardening intervention programs for older adults are effective in improving vegetable and fruit consumption in the population [154]. Moreover, improving access to local food markets that offer MD products may enhance older adults’ ability to maintain a nutritionally adequate dietary pattern. The relationship between the availability of healthy food options within residential neighborhoods and the prevalence of disability and cardiovascular diseases has been investigated [155,156]; however, further research is needed to study its actual impact, with particular emphasis on MD foods and related environmental factors.

Adherence to the MD principles entails a healthy lifestyle that has been proven to enhance self-realization, control, life enjoyment, and autonomy, essential elements for healthy aging [157]. MD retains a high socio-cultural food value [158] as it is closely linked to the traditions and the cultural, social, and economic value that food has for Mediterranean people. Eating is not just about meeting nutritional and energy needs but also encompasses the care of food preparation, the pleasure in convivial moments, and the enjoyment of family meals. Mediterranean meals are an opportunity for social exchange and communication [158], which reduces loneliness, thus promoting psychological well-being and general health [159]. These are central aspects in elderly nutrition; indeed, eating is strictly connected to the psycho-affective dimension in which the meal is served and strongly impacts food intake and meal pleasure [160].

Public interventions aimed at providing affordable and accessible MD foods could positively impact the health of older populations and support broader social sustainability. Indeed, some strategies could be adapted to individuals affected by serious mental illnesses to encourage healthier eating habits [161]. These interventions involved motivational elements, such as familiarization with local farmers’ markets and guided visits to specific grocery stores. Such initiatives could be delivered through seminars and community events led by healthcare professionals, emphasizing diet, lifestyle, health, and conviviality—core pillars of the MD that contribute to promoting well-being and enhancing quality of life in older adults.

### Barriers and Policy Opportunities for Supporting Mediterranean Diet Adherence in Older Populations

The accessibility of MD foods for older adults is often influenced by social and economic constraints. Irz et al., in 2014 [162], investigated this issue across four European countries (Italy, Sweden, the United Kingdom, and Finland) and reported no positive association between nutritional health and economic resources. According to the authors, this finding may be considered encouraging, as it underscores the potential of public health policies, particularly those focused on information and education, over purely economic measures. Informational and educational interventions aimed at raising awareness of the health benefits of a balanced diet represent an effective strategy to enhance dietary quality among older populations.

A 12-year scientific literature review (2013–2024) by Colaprico et al. in 2024 [163] further highlighted the health benefits associated with adopting the MD, indicating potential savings in healthcare expenditures and therefore suggesting that national public health programs should promote policies that support and encourage this lifestyle.

Additional research by Alves and Perelman, in 2022 [164], reported a general increase in MD adherence among the European elderly population, mainly due to a reduction in animal protein consumption and an increase in legume intake. The authors speculated that this trend—more evident among affluent, educated, and healthy individuals—may be related to a more favorable economic background that enables greater consideration of health and environmental factors in food choices. However, these positive developments may also exacerbate dietary and health inequalities, highlighting the need for public interventions to mitigate this risk and, more specifically, to address social deprivation [165].

A rational and evidence-based use of institutional catering can also play a significant role in supporting older adults’ adherence to the MD within residential settings, as demonstrated by the HECTOR project [166]. Subsequent studies have explored approaches to influence food choices among older adults toward more plant-based options, in line with the core principles of the MD [167]. Among these, the implementation of a nudging strategy—specifically, the introduction of a “dish of the day” option in catering services targeting older consumers in Denmark, France, Italy, and the United Kingdom—has been proven effective, with sensory appreciation emerging as one of the primary determinants of food selection. Overall, the nudging approach appears to be a promising strategy for fostering sustainable and health-oriented dietary behaviors in institutional catering environments.

Furthermore, Zhou et al., in 2018 [168], in a broad overview of interventions promoting healthy eating among older adults in Europe and other countries, reported that dietary education, meal-service interventions, and multicomponent strategies are effective in increasing fruit and vegetable intake, diversifying food choices, and improving overall nutritional status. However, a recent literature review by Turner et al. in 2023 [169] noted that most research on lifestyle–diet–health interconnections focuses on developing interventions and evaluating their effects on clinical markers, while much less attention is devoted to translating research outcomes into population-level implementation. These findings highlight a clear need for stronger collaboration between researchers, who generate evidence and devise intervention strategies, and the stakeholders, responsible for implementing these measures in real-world settings, to enhance population well-being.

## 7. Strengths and Limitations

The present study is a narrative review that examines the main benefits associated with adherence to the Mediterranean dietary pattern and lifestyle. Its key strength lies in the interdisciplinary approach adopted, which combines socio-cultural perspectives with biochemical and sensory aspects. Therefore, despite consistent evidence linking Mediterranean dietary patterns to improved aging trajectories, several limitations persist. Definitions of adherence and scoring systems for MD indices vary considerably, introducing heterogeneity across cohorts. Most data derive from observational designs subject to residual confounding, while long-term randomized trials in older adults with multimorbidity are scarce. Pragmatic studies in nursing homes and long-term care facilities are urgently needed to evaluate real-world applicability. Future research should standardize exposure metrics and integrate biomarkers of adherence and functional outcomes.

## 8. Conclusions

Due to the increase in life expectancy, public health services are becoming increasingly involved in improving the quality of life and extending the number of healthy years. This goal is pursued not only through general healthcare provision but also through specific preventive actions, such as creating physical and institutional environments that support a happy and healthy life [157]. In this context, eating habits play a crucial role, as an adequate diet was effective in maintaining good mental health in old age [170,171]. Owing to substantial scientific advances, it is now widely accepted that healthy aging in humans is closely associated with proper nutrition, which often entails avoiding unhealthy foods [172]. This review showed how the MD principles represent a comprehensive and evidence-based framework for mitigating age-related health decline and promoting well-being in later life while simultaneously fostering international scientific collaboration on the influence of traditional dietary practices on population health outcomes [173].

## Figures and Tables

**Table 1:** Plant-based foods typical of the MD and their bioactive compounds, mechanisms, and evidence intensity for healthy aging. Evidence type is indicated as “Strong” = supported by multiple large cohorts and meta-analyses or long-term RCTs; “Moderate” = consistent observational data and some RCTs/short-term interventions; “Translational” = mainly mechanistic, in vitro/animal or short-term human studies. The following abbreviations are used: “CVD”: Cardiovascular Disease; “T2D”: Type 2 Diabetes; “MUFA”: Monounsaturated Fatty Acids; “PUFA”: Polyunsaturated Fatty Acids; “BP”: Blood Pressure; “Mg”: Magnesium; “RCT”: Randomized Controlled Trial; “LDL”: Low-Density Lipoprotein.

Food Group	Main Bioactive Compounds	Dietary Doses/Servings	Main Geriatric Outcomes/Mechanisms	Evidence Type	Evidence Intensity	Key References
Whole grains	Dietary fiber, β-glucans, phenolic acids (ferulic, caffeic, p-coumaric), lignans, tocols, phytosterols	3–6 servings/day (~90–180 g/day)	Reduced CVD and T2D risk; improved lipid and glucose metabolism; antioxidant and microbiota effects	Meta-analyses, multicenter cohorts (clinical and observational)	Strong	[30,31,32,41]
Legumes	Plant proteins, fermentable fiber, isoflavones, saponins, flavonoids, bioactive peptides	3–4 servings/week (~100–150 g cooked per serving)	Improved glycemic and lipid control; antioxidant and anti-inflammatory actions	RCTs + cohorts	Moderate	[42,43]
Tomato and tomato by-products	Lycopene, polyphenols (quercetin, naringenin), phytosterols, polyamines	1–2 servings/day (~150–200 g fresh or 80 g canned/tomato paste)	Antioxidant and anti-inflammatory; vascular and metabolic benefits; antithrombotic activity	Meta-analysis + systematic reviews + translational studies	Moderate (translational)	[44,45,46]
Leafy vegetables and cucurbits	Carotenoids (lutein, zeaxanthin), folates, vitamin K1, glucosinolates, saponins, fiber	No doses indicated in selected papers	Visual and cognitive protection; anti-inflammatory; improved glycemic and lipid control	Prospective cohorts + narrative review	Moderate	[47,48]
Citrus fruits	Flavanones (hesperidin, naringin), polymethoxylated flavones, vitamin C	1–2 servings/day (~150–200 g fruit or 125 mL juice)	Endothelial protection, anti-inflammatory and bone-supporting effects	RCTs + systematic reviews (clinical)	Moderate	[49,50,51,52,53]
Allium species	Organosulfur compounds (allicin, S-allyl cysteine), phenolics (rutin, quercetin)	1–2 cloves garlic/day (3–5 g), or ½ cup chopped onion (~75 g)	BP and cholesterol reduction; antimicrobial and immunomodulatory effects	Meta-analyses + short-term RCTs	Moderate	[54,55,56]
Nuts	MUFAs, PUFAs, tocopherols, phytosterols, polyphenols (ellagitannins, flavan-3-ols), L-arginine, Mg	1 serving/day (~30 g)	Reduced LDL oxidation, inflammation, improved endothelial and cognitive function	Meta-analyses + long-term RCTs + systematic and narrative reviews	Strong	[57,58,59]
Capers, herbs and spices (Mediterranean seasonings)	Terpenoids, essential oils, polyphenols (e.g., rosmarinic acid, apigenin)	No doses indicated in selected papers	Improved cerebral blood flow, modulation of cholinergic neurotransmission, reduced neuroinflammation, cognitive and vascular support	Mechanistic and preclinical studies (translational)	Limited (preclinical)	[60,61,62,63]

**Table 2:** Mediterranean dietary patterns and synergistic effects on healthy aging. The following abbreviations are used: “CVD”: Cardiovascular Disease; “RCT”: Randomized Controlled Trial.

Dietary Pattern/Study	Key Components	Main Findings Related to Aging	Evidence Type	Key References
Mediterranean diet overall	High plant-based foods, olive oil, moderate wine	Reduced all-cause mortality, CVD, frailty, cognitive decline	Meta-analyses, prospective cohorts	[123,124]
NU-AGE multicenter trial	Personalized Mediterranean-like diet, micronutrient adequacy	Improved gut microbiota composition reduced frailty and improvements in selected immune and metabolic biomarkers (inflammaging-related signatures). Multicenter RCT across 5 European countries; generalizability limited by tailored intervention and 1-year duration	Multicenter RCT	[125,126]
Polyphenol-rich patterns	High intake of fruits, vegetables, tea, cocoa, red wine	Reduced oxidative stress, inflammation, and vascular aging	Systematic reviews	[105]
